# The correlation between multi-mode ultrasonographic features of breast cancer and axillary lymph node metastasis

**DOI:** 10.3389/fonc.2024.1433872

**Published:** 2024-11-04

**Authors:** Shijian Xu, Qi Wang, Zhe Hong

**Affiliations:** Department of Ultrasound, The Fifth Affiliated Hospital of Sun Yat-sen University, Zhuhai, China

**Keywords:** breast cancer, axillary lymph node, shear wave elastography, “stiff rim” sign, ultrasound

## Abstract

**Objective:**

This study aimed to explore the correlation between multi-mode ultrasonographic features of breast cancer and axillary lymph node metastasis.

**Method:**

A total of 196 patients with surgically confirmed breast cancer between September 2019 and December 2023 were included. Data on preoperative B-mode ultrasound (US), color Doppler, and shear wave elastography (SWE) features of breast cancer masses were collected and analyzed to determine their correlation with axillary lymph node metastasis. The area under the receiver operating characteristic curve (AUC) of B-mode US, color Doppler, SWE, and the multi-mode predictive model for evaluating axillary lymph node metastasis were compared.

**Results:**

Among the 196 patients, 70 had positive axillary lymph nodes, while 126 had negative axillary lymph nodes. There was no significant difference in the color features between the negative and positive axillary lymph node groups. Multifocality/multicentricity, architectural distortion, microcalcifications, and the “stiff rim” sign in SWE were identified as independent risk factors to predict axillary lymph node metastasis according to binary logistic regression analysis. The AUC of the predictive model based on these independent risk factors was 0.803 (95% CI: 0.739–0.867), which was significantly higher than that of B-mode US or SWE alone.

**Conclusion:**

Multifocality/multicentricity, architectural distortion, microcalcifications, and the “stiff rim” sign in SWE were found to be valuable for predicting axillary lymph node metastasis in patients with breast cancer. The predictive model developed in this study, combining the multi-mode ultrasonographic features of breast cancer masses, could serve as a noninvasive and convenient method to predict axillary lymph node status. This approach could aid in clinical decision-making and individualized treatment to improve the prognosis of breast cancer patients.

## Introduction

Breast cancer ranks among the most prevalent malignancies worldwide, surpassing even lung and colorectal cancers in incidence among women (1). The prognosis of breast cancer hinges significantly on various factors, particularly the presence of metastasis in the axillary lymph nodes, which is crucial for both clinical decision-making and prognostic assessment (2). Mammography is not valuable in evaluating axillary lymph node status. MRI has higher specificity, but it is not convenient and is time-consuming. Ultrasound offers a noninvasive, convenient, and radiation-free alternative. However, distinguishing metastatic axillary lymph nodes from reactive ones presents a challenge, with reported sensitivities ranging from 22.5% to 87.1% and specificities from 44.7% to 100% (3).

Elastography, which includes both qualitative and quantitative approaches, plays an important role in differentiating benign from malignant breast masses. Although studies have highlighted the utility of quantitative shear wave elastography (SWE) parameters in predicting axillary lymph node metastasis, there is ongoing debate over the optimal cutoff values (4–7). Evans et al. identified *E*_mean_ as an independent predictor, with axillary lymph node metastasis rates of 7% when *E*_mean_ was <50 kPa and 41% when >150 kPa (8). Additionally, researchers have explored SWE’s application directly to axillary lymph nodes, with findings such as Eratio serving as an independent risk factor (9). Nevertheless, the anatomical intricacies of the axilla and lymph node locations pose challenges in obtaining perfect SWE images.

Given the complexity of evaluating axillary lymph node status in breast cancer, our study aimed to assess the predictive value of B-mode ultrasound (US), color Doppler, and SWE in this context. Furthermore, we endeavored to develop a predictive model for axillary lymph node status in breast cancer patients.

## Materials and methods

### Patients

**Figure 1:**
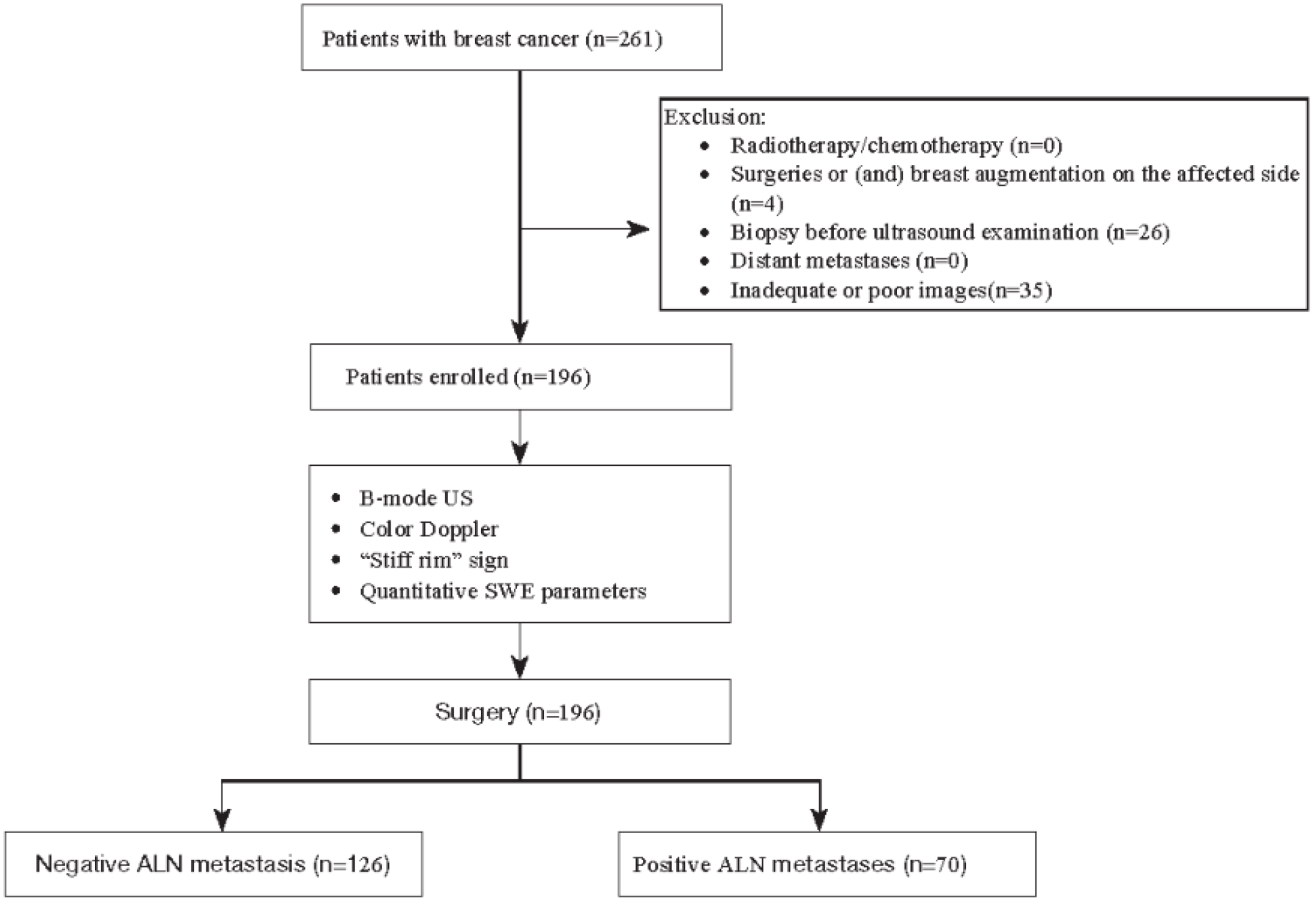
Flowchart of this study. SWE, shear wave elastography; ALN, axillary lymph node.

This retrospective study was approved by the institutional review board, and informed consent was waived. The inclusion criteria were as follows: (1) pathological results were confirmed as breast cancer by surgery, (2) maximum diameter of mass ≤5 cm, and (3) radical mastectomy and axillary lymph node dissection were performed within 2 weeks after ultrasound examination. The exclusion criteria were as follows: (1) patients who had received radiotherapy/chemotherapy before ultrasound examination, (2) patients who had received other surgeries or (and) breast augmentation on the affected side before, (3) patients who had undergone breast mass biopsy before ultrasound examination, or (4) patients who had distant metastases. From September 2019 to December 2023, a total of 261 women diagnosed with breast cancer via surgery were initially enrolled. Following the exclusion of 26 patients who underwent biopsy prior to ultrasound, 35 with inadequate or poor sonographic images, and four who had undergone other surgeries or breast augmentation, the final analysis included 196 patients confirmed to have breast cancer through surgical pathology (**Figure 1**).

### Ultrasound examinations

All B-mode US examinations, color Doppler, and SWE examinations of breast cancer masses were conducted by two radiologists with 5–10 years of experience in ultrasound examination, using the Aixplorer^®^ system (Supersonic Imagine, Aix en Provence, France) with a 4–15-MHz linear array transducer. Images of breast masses recording B-mode US examinations, color Doppler, and SWE features were stored and analyzed. In cases where images were disputed, the sonographers engaged in discussions to reach a consensus on the results.

### B-mode US and color examinations

The patients were positioned in a supine position with their arms naturally raised to fully expose both breasts and axillas. B-mode US examination features include maximal diameter of breast mass, location, multifocality/multicentricity, orientation, margin characteristics, microcalcifications within the mass, and architectural distortion according to the 2013 edition of the Breast Imaging Reporting and Data System (BI-RADS) (10). With a scale of 4 cm/s in color Doppler mode, the breast masses were classified as hypovascular and hypervascular. Hypovascular masses were defined by the absence of flow or the presence of one or two pixels containing flow (usually less than 1 mm in diameter) in or around the mass, while hypervascular masses were characterized by the presence of at least one main vessel in or around the mass (11).

### SWE examinations

The “stiff rim” sign has been reported as a characteristic feature of breast cancer (12). It is defined as an area of increased stiffness in peripheral compared to the center of a breast mass. With a scale of 180 kPa, the patients were instructed to hold their breath, and stable images with good quality and no obvious artifacts were obtained. The presence of the “stiff rim” sign at 180 kPa was observed and, if absent, the threshold was lowered to 80 kPa to observe its presence. Quantitative SWE measurements of breast masses and surrounding normal tissue were obtained via the region of interest (ROI) with Q-Box. The first 2 × 2 mm^2^ Q-Box was placed at the hardest area inside or at the edge of the breast mass, obtaining the maximum (*E*_max_), mean (*E*_mean_), minimum (*E*_min_), and standard deviation (SD) values. Subsequently, the second region of interest (Q-BOX2) of the same size was placed in the surrounding normal breast tissue at the same depth as Q-BOX1 to obtain the elasticity ratio (Eratio) between the hardest area of the breast mass and the surrounding normal tissue. Quantitative SWE parameters were collected five times and the average obtained.

### Statistical analysis

Statistics analysis was performed using SPSS 26.0 (IBM Corp., Armonk, NY, USA). Mean and standard deviations (SD) for quantitative variables were used, and chi-square or Fisher exact tests were used for categorical variables. Receiver operating characteristic curves (ROC) were constructed to predict axillary lymph node metastasis based on B-mode US, color SWE, and combined features of breast masses. The sensitivity, specificity, positive predictive value (PPV), negative predictive value (NPV), accuracy, and area under the receiver operating characteristic curve (AUC) were compared. A *P* value of <0.05 was considered statistically significant.

## Results

Of the 196 enrolled breast cancer patients, 126 (64.3%) were confirmed to have negative axillary lymph nodes, while 70 (35.7%) were positive. There was no significant difference in age between the positive axillary lymph node group (48.7 ± 10.6 years) and the negative axillary lymph node group (48.7 ± 9.6 years) (*P*>0.05).

### Comparison of B-mode US and color features between positive and negative axillary lymph node groups

In the B-mode US features of breast cancer, the size of the breast mass was significantly larger in the positive axillary lymph node group (2.96 ± 1.24 cm) compared to the negative axillary lymph node group (2.22 ± 1.03 cm) (*P* < 0.05). Multifocality/multicentricity, microcalcifications within the mass, and architectural distortion were more likely to occur in patients with axillary lymph node metastasis (*P*<0.05). However, there were no significant differences in the location, orientation, or margin characteristics between the axillary lymph node positive and negative groups (*P* > 0.05) (**Table 1**). Neither color pattern nor Vmax and RI were correlated with axillary lymph node metastasis (**Table 2**).

**Table 1:** Correlation between B-mode ultrasonographic features of breast cancer and axillary lymph node metastasis.

	Positive axillary lymph node	Negative axillary lymph node	*P*
Size	2.96 ± 1.24 cm	2.22 ± 1.03 cm	0.008
Location			
Outer upper quadrant	33 (16.8%)	50 (25.5%)	
Outer lower quadrant	9 (4.6%)	15 (7.7%)	
Inner upper quadrant	10 (5.1%)	23 (11.7%)	
Inner lower quadrant	4 (2.0%)	7 (3.6%)	
Center	31 (15.8%)	14 (7.1%)	
Multifocality/multicentricity			0.001
Presence	22 (11.2%)	16 (8.2%)	
Absence	110 (56.1%)	48 (24.5%)	
Orientation			0.234
Parallel	45 (23.0%)	70 (35.7%)	
Not parallel	25 (12.7%)	56 (28.6%)	
Margin			0.149
Indistinct, angular, microlobulated, or spiculated	58 (29.6%)	93 (47.4%)	
Circumscribed	12 (6.1%)	33 (16.8%)	
Microcalcifications in mass			0.002
Presence	37 (18.9%)	42 (21.4%)	
Absence	33 (16.8%)	84 (42.9%)	
Architectural distortion			<.001
Presence	37 (18.9%)	33 (16.8%)	
Absence	33 (16.8%)	93 (47.4%)	

**Table 2:** Correlation between the color features of breast cancer and axillary lymph node metastasis.

	Positive axillary lymph node	Negative axillary lymph node	*P*
Hypovascular	9 (4.6%)	15 (7.7%)	0.845
Hypervascular	61 (31.1%)	111 (56.6%)	
RI	0.78 ± 0.13	0.76 ± 0.12	0.266
Vmax	18.1 ± 13.3	16.3 ± 10.0	0.205

### Comparison of SWE features between positive and negative axillary lymph node group

There were 66 patients with positive axillary lymph node that exhibited the “stiff rim” sign in SWE, which was significantly more than in the axillary lymph node-negative group (*P* < 0.05). However, there were no correlations between quantitative parameters and “stiff rim” sign metastasis in this study (*P* > 0.05) (**Table 3**).

**Table 3:** Correlation between the SWE features of breast cancer and axillary lymph node metastasis.

	Positive axillary lymph node	Negative axillary lymph node	*P*
“Stiff rim” sign	66 (33.7%)	96 (49.0%)	0.001
No-“stiff rim” sign	4 (2.0%)	30 (15.3%)	
*E*_mean_ (kPa)	153.61 ± 57.89	145.05 ± 65.64	0.258
*E*_min_ (kPa)	110.57 ± 59.81	105.87 ± 58.82	0.898
*E*_max_ (kPa)	182.62 ± 61.88	166.98 ± 73.55	0.141
SD (kPa)	20.34 ± 10.52	17.26 ± 12.24	0.87
Eratio	19.63 ± 11.28	15.49 ± 11.04	0.99

### Multivariate analysis of B-mode US, color Doppler, and SWE to predict axillary lymph node metastasis

Based on the univariate analysis results, size, multifocality/multicentricity, microcalcifications within the mass, architectural distortion, and “stiff rim” sign were significantly different between the axillary lymph node positive and negative group. According to binary logistics regression analysis, multifocality/multicentricity, microcalcifications within the mass, architectural distortion, and “stiff rim” sign were independent risk factors to predict axillary lymph node metastasis (**Table 4**). The predictive model was constructed based on binary logistics regression analysis results: Logit(*P*) = 0.874 + 1.816 × multifocality/multicentricity + 0.938 microcalcifications + 1.145 × architectural distortion + 1.754 × “stiff rim” sign.

**Table 4:** Multivariate analysis of B-mode US, color Doppler, and SWE to predict axillary lymph node metastasis.

Variable	β	Odds ratio (95% CI)	*P*
	0.874	–	–
Multifocal/Multicentric	1.816	1.163 (1.064–1.414)	<0.001
Microcalcifications	0.938	1.391 (1.193–1.796)	0.010
Architectural Distortion	1.145	1.318 (1.152–1.664)	0.002
“Stiff rim” sign	1.754	1.173 (1.052–1.578)	0.004

### Comparison of diagnostic performance between independent risk factors and combined B-mode US features and predictive model

**Figure 2:**
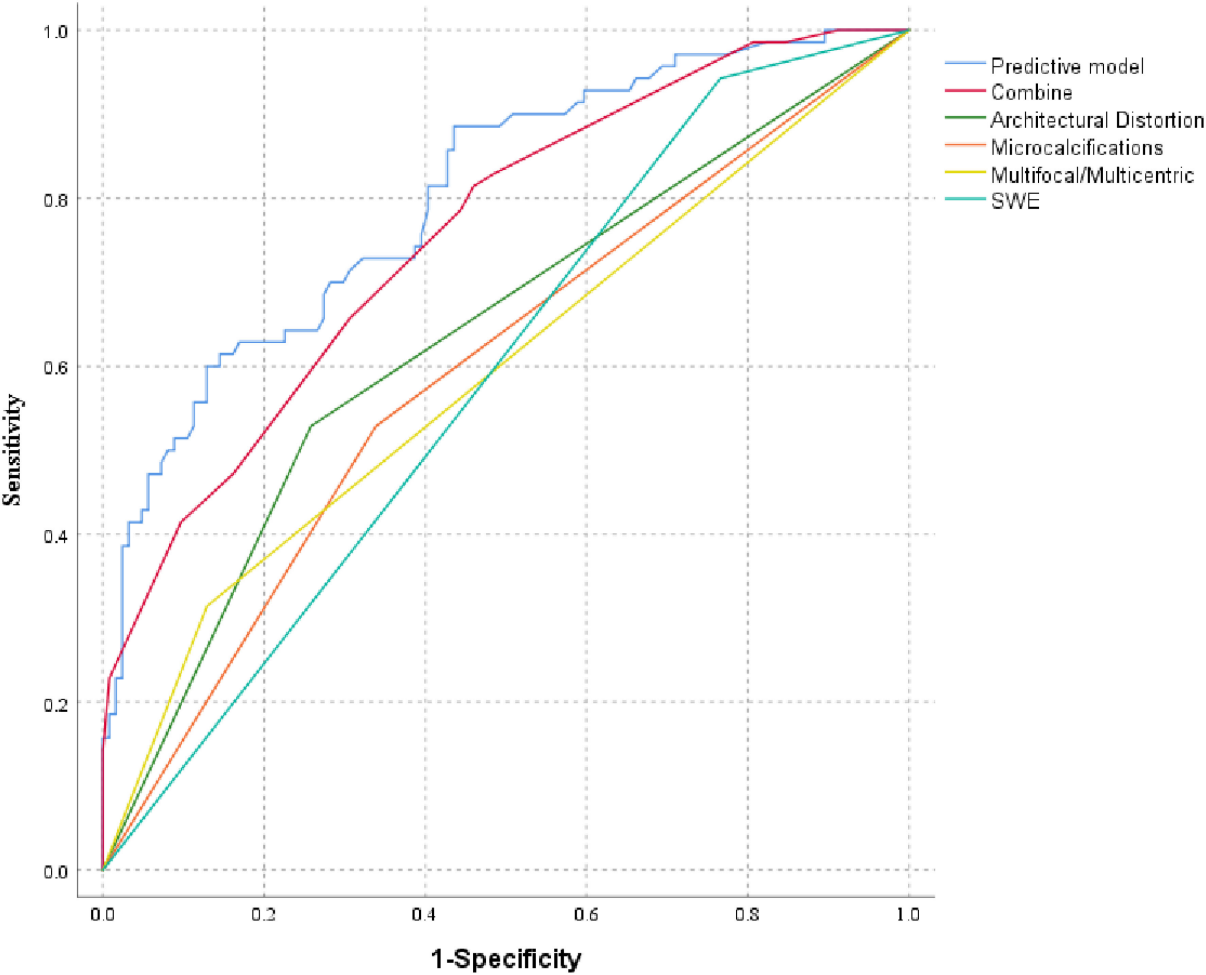
Receiver operating characteristic curves of independent risk factors, B-mode US, and the predictive model; “combine” included B-mode US features, multifocality/multicentricity, microcalcifications within the mass, and architectural distortion.

**Figure 3:**
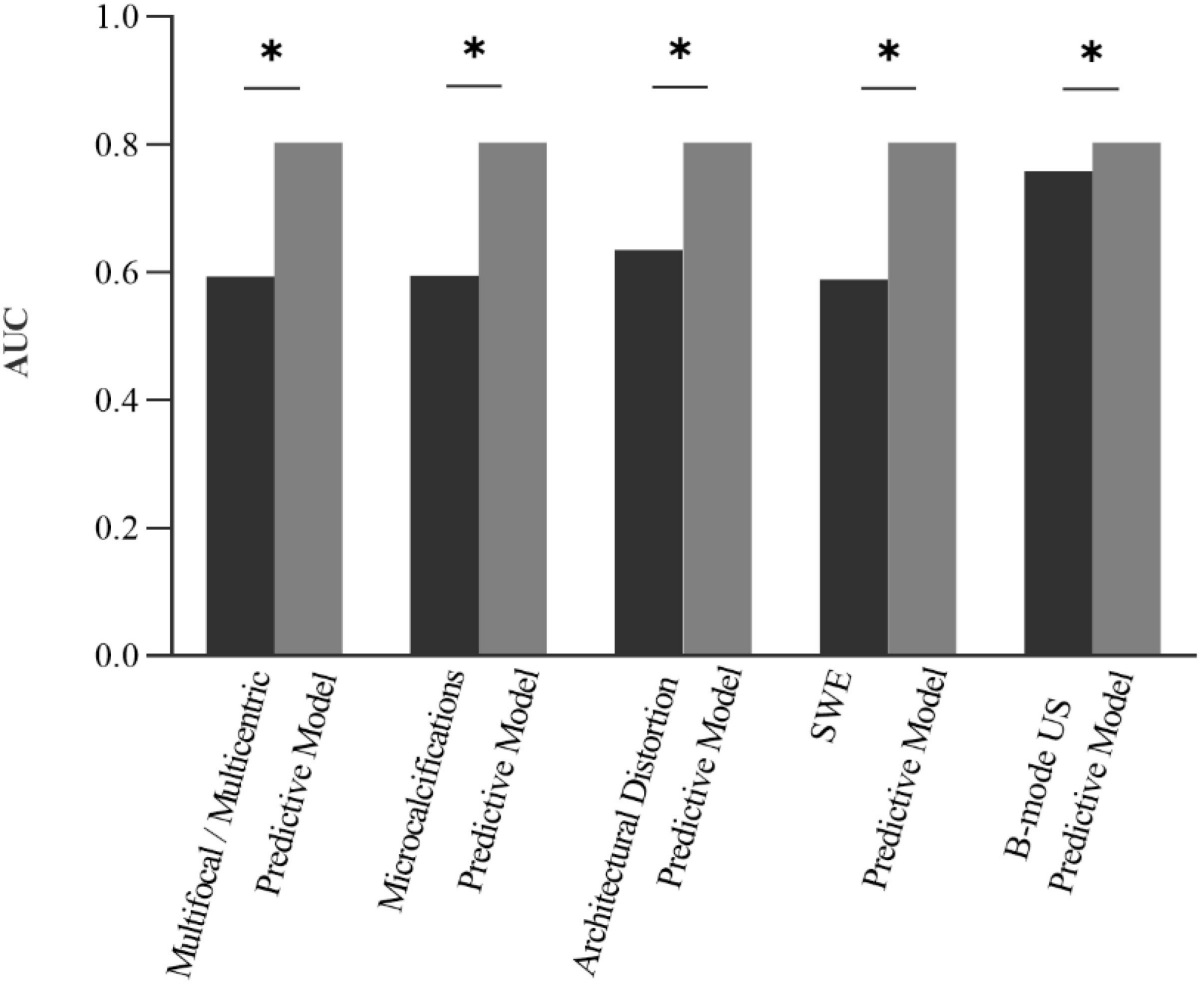
The AUC of the predictive model was significantly higher than the other features individually and the combined B-mode US features. * means the difference is significant.

A receiver operating characteristic curve (ROC) was plotted to evaluate the independent risk factors and combined B-mode US features and predictive model. The AUC of the predictive model was 0.803 (95% CI: 0.739–0.867), which was significantly higher than the others (**Figures 2**, **3**).

## Discussion

Our study demonstrates that the combination of B-mode US features and the “stiff rim” sign is valuable for predicting axillary lymph node metastasis in breast cancer patients. Additionally, we successfully established a predictive model for axillary lymph node metastasis in these patients, achieving the highest area under the curve (AUC) of 0.803 (95% CI: 0.739–0.867) compared to B-mode US features alone. Notably, we found no significant correlation between color features and axillary lymph node metastasis.

Assessing the status of axillary lymph nodes is crucial to guide clinical decision-making and prognosis in breast cancer patients, making it beneficial for preoperative treatment. US, mammography, and MRI are important preoperative examinations for axillary lymph node status of breast cancer (13). While MRI has higher specificity compared to US and mammography, its sensitivity remains low. Conventional ultrasound evaluation of axillary lymph nodes is mainly based on morphological changes such as lymph node long axis/short axis (L/S) <2, heterogeneous cortical thickening, disappearance of the lymph node hilum, peripheral or mixed blood flow, and absence of microcalcifications in axillary lymph nodes (14). However, even with relatively high specificity, there were considerable differences in the reported sensitivities (15), highlighting the need for additional valuable information for the preoperative assessment of lymph node status in breast cancer patients.

In recent years, there has been growing interest in predicting axillary lymph node metastasis using quantitative SWE in breast cancer patients. Evans et al. reported that mean stiffness on SWE was an independent risk factor to predict axillary lymph node metastasis in breast cancer patients. When *E*_mean_ was <50 kPa, the axillary lymph node metastasis rates was 7%, but when *E*_mean_ was >150 kPa, the axillary lymph node metastasis rate was 41% (8). Wen et al. reported that *E*_max_ had the best diagnostic performance in predicting axillary lymph node metastasis among all quantitative SWE parameters (6), while Jiang et al. reported that Eratio had the best diagnostic performance (7). The best quantitative SWE parameters to predict axillary lymph node metastasis varied across different studies.

**Figure 4:**
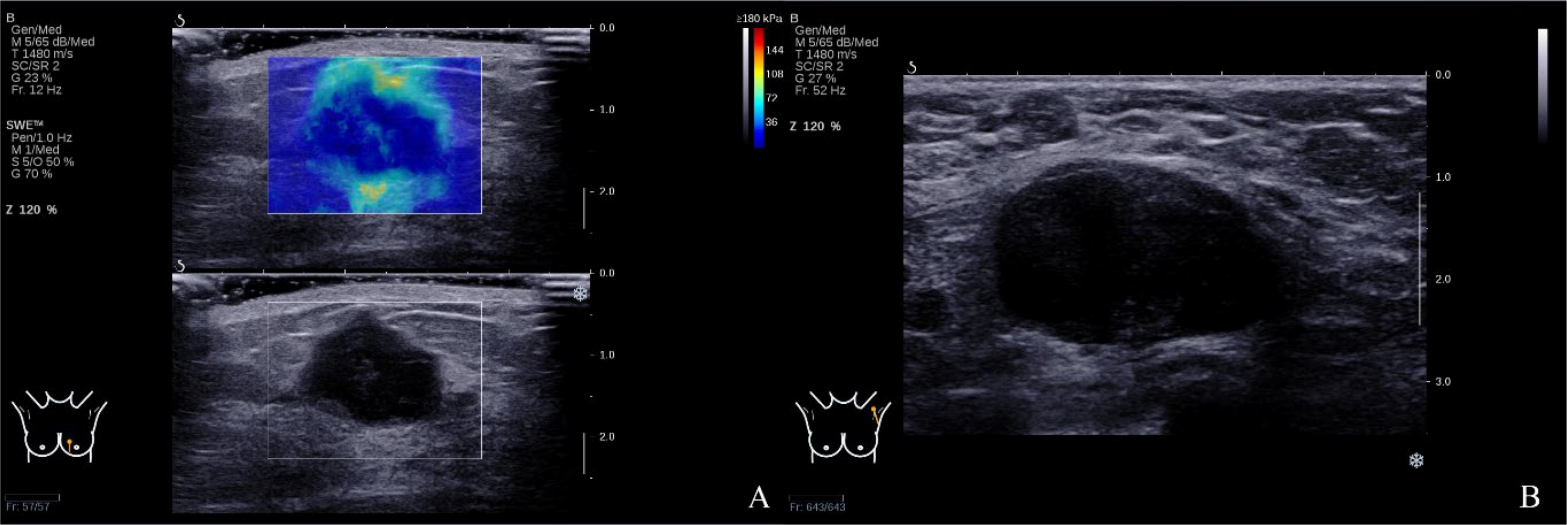
**(A)** The left breast mass SWE showed the “stiff rim” sign. **(B)** The left axillary lymph node was pathologically confirmed as metastasis.

**Figure 5:**
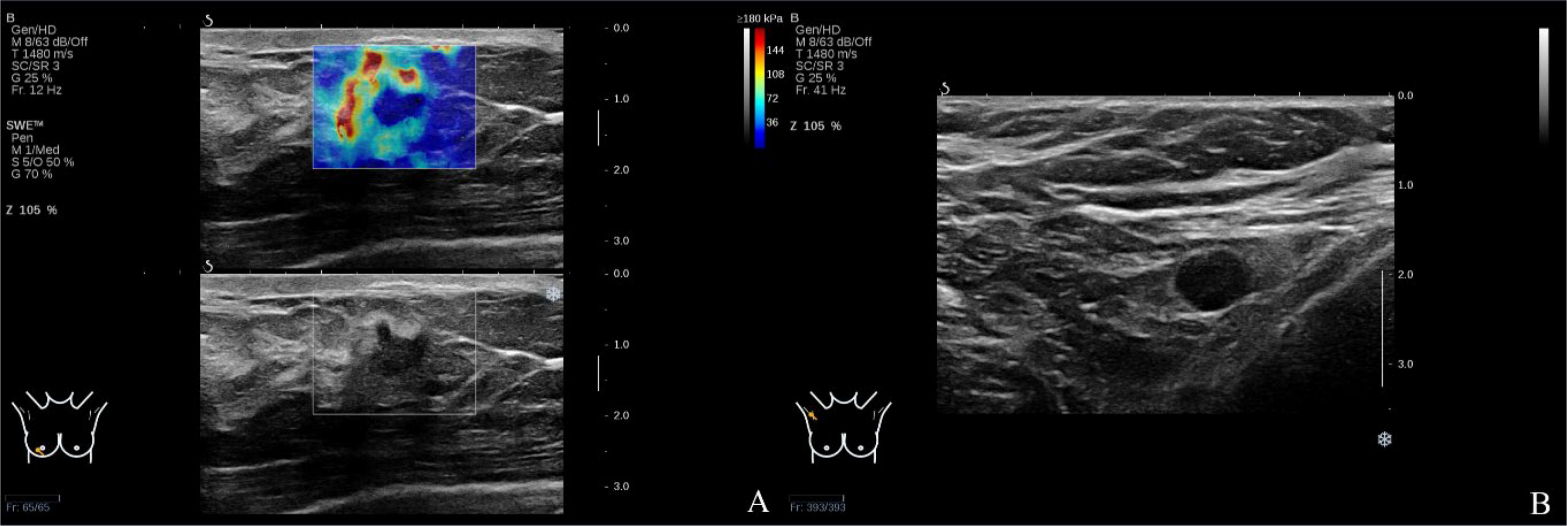
**(A)** The right breast mass SWE showed the “stiff rim” sign. **(B)** The right axillary lymph node was pathologically confirmed as metastasis.

Lin et al. reported seven color patterns in qualitative SWE presenting as no findings, vertical stripes, spot pattern, rim of stiffness pattern, colored lesion pattern, void center pattern, and horseshoe pattern. No findings, vertical stripes, and spot pattern were considered related to benign breast masses, and rim of stiffness pattern, colored lesion pattern, void center pattern, and horseshoe pattern were considered related to malignant breast masses (16). Among all the malignant signs, “stiff rim” sign was considered a specific manifestation in qualitative SWE (12). The stromal reaction of breast cancer tumor cells leads to increased fibroblast growth toward the tumor edge, increased cross-linking of collagen fibers, and increased tissue hardness. “Stiff rim” sign was regarded as the infiltration of cancer cells into the interstitial tissues or a desmoplastic reaction which is an independent prognostic factor predicting poor prognosis. The stromal reaction of tumor cells also enhances the activity of phosphoinositide 3-kinase, which may induce tumor metastasis (17–19). However, few studies have investigated the relationship between “stiff rim” sign and axillary lymph node metastasis. Hence, we tried to find if there is any correlation between “stiff rim” sign and axillary lymph node metastasis in this study. Our study found that “stiff rim” sign is more prevalent in patients with positive axillary lymph nodes (**Figures 4**, **5**). Out of 162 patients showing the “stiff rim” sign, 66 had positive axillary lymph nodes, while only four patients with positive axillary lymph nodes did not show the “stiff rim” sign on SWE, indicating a significant correlation with axillary lymph node metastasis. The result was consistent with a previous study (20). A predictive model was established to predict axillary lymph node metastasis in patients with breast cancer according to the binary logistic regression analysis of B-mode US features and SWE. The model had best AUC 0.803 (95% CI: 0.739–0.867) compared to B-mode US features or the “stiff rim” sign alone. The predictive model of multi-mode ultrasonographic features could be a noninvasive and convenient method to predict axillary lymph node metastasis in patients with breast cancer.

There were several limitations in our study. Firstly, it was a retrospective study, which involved some inevitable bias. Secondly, the color features included in this study were not obtained by microvascular Doppler ultrasound technique. Thirdly, it was a single-center study that did not have a large sample size; thus, multicenter studies with larger samples should be performed in the future.

## Conclusions

Multifocality/multicentricity, architectural distortion, microcalcifications, and the “stiff rim” sign in SWE were found to be valuable to predict axillary lymph node metastasis in patients with breast cancer. Furthermore, our predictive model, incorporating multi-mode ultrasonographic features, offers a noninvasive and convenient approach to predict axillary lymph node metastasis, thereby aiding clinical decision-making and individualized treatment to enhance the prognosis of breast cancer patients.

## Data Availability

The raw data supporting the conclusions of this article will be made available by the authors, without undue reservation.
